# Community vulnerability to the COVID-19 pandemic: A narrative synthesis from an ecological perspective

**DOI:** 10.7189/jogh.12.05054

**Published:** 2022-12-03

**Authors:** Qiuyan Liao, Meihong Dong, Jiehu Yuan, Wendy Wing Tak Lam, Richard Fielding

**Affiliations:** School of Public Health, LKS Faculty of Medicine, The University of Hong Kong, Hong Kong Special Administrative Region, China

## Abstract

**Background:**

We aimed to conduct a narrative synthesis of components and indicators of community vulnerability to a pandemic and discuss their interrelationships from an ecological perspective.

**Methods:**

We searched from PubMed, Embase, Web of Science, PsycINFO, and Scopus (updated to November 2021) for studies focusing on community vulnerability to a pandemic caused by novel respiratory viruses on a geographic unit basis . Studies that reported the associations of community vulnerability levels with at least one disease morbidity or mortality outcome were included.

**Results:**

Forty-one studies were included. All were about the COVID-19 pandemic. Suitable temperature and humidity environments, advanced social and human development (including high population density and human mobility, connectivity, and occupations), and settings that intensified physical interactions are important indicators of vulnerability to viral exposure. However, the eventual pandemic health impacts are predominant in communities that faced environmental pollution, higher proportions of socioeconomically deprived people, health deprivation, higher proportions of poor-condition households, limited access to preventive health care and urban infrastructure, uneven social and human development, and racism. More stringent social distancing policies were associated with lower COVID-19 morbidity and mortality only in the early pandemic phases. Prolonged social distancing policies can disproportionately burden the socially disadvantaged and racially/ethnically marginalized groups.

**Conclusions:**

Community vulnerability to a pandemic is foremost the vulnerability of the ecological systems shaped by complex interactions between the human and environmental systems.

**Registration:**

PROSPERO (CRD42021266186).

COVID-19 afflicted populations globally. Some communities have been disproportionally impacted [1]. A community is a group of mutually interdependent people residing within a defined geographic area whose routine interactions are integrated within the shared socio-cultural systems and environments [2]. A pandemic’s impact on a community depends on community vulnerability – that is, the degree to which it is susceptible to and adaptable to adverse effects [3]. Community vulnerability is a dynamic phenomenon involving complicated interactions between the biophysical and social processes within the ecological systems [3]. Hence, metrics for community vulnerability can be complicated and difficult to quantify. Nevertheless, efforts to measure and map community vulnerability to a pandemic have been widespread, aiming to optimize the allocation of finite resources and adapt policymaking for pandemic preparedness, response, mitigation, and recovery.

Considerable research has emerged focusing on measuring community vulnerability to the COVID-19 pandemic [4-12]. Most portrays vulnerability as the absence of entitlements (resources available to individuals) or entitlement failure [13]. A main application of the entitlement theory is the social vulnerability index (SVI) proposed by the United States Centre for Disease Control and Prevention (CDC) which is assessed using four themes – socioeconomic status, household composition and disability, minority status and language, and housing and transportation [4-7]. The Surgo Foundation further expanded the CDC’s SVI to additionally encompass population health conditions, health care capacity, and risky occupational environments, to construct a COVID-19 community vulnerability index (CCVI) [8]. Some studies extended the SIV and CCVI to additionally include social development [10,14], urban built environments [9,10], natural environments [11,12] and COVID-19 control policies [15], to provide a more comprehensive evaluation of community vulnerability to COVID-19.

Evolving insights underline the importance of system-oriented analysis of vulnerability [3]. Community vulnerability is fundamentally the vulnerability of the ecological systems that comprise human and environmental systems and their interactions and interdependence [2]. Devastation occurs when the ecological systems cannot absorb the disturbances and multiple stresses caused by the pandemic [16]. A system-oriented analysis focuses on not merely what components are important for determining community vulnerability, but also how they interact and depend on each other, and thus facilitate the understanding about the mechanisms and processes of community vulnerability [3,17]. The framework of coupled human-environment system for vulnerability analysis represents one important conceptual advance for system-oriented vulnerability analysis [18,19]. Based on the framework, community vulnerability is portrayed as the vulnerability of the dynamic coupled human-environment systems, with stress-induced feedbacks, institutional decisions, and human behaviours acting back upon the systems themselves [3,17].

We conducted a narrative literatures synthesis in response to the growing studies on community vulnerability relevant to a pandemic. A narrative synthesis approach was used due to the high heterogeneity in metrics for quantifying community vulnerability, geographic units of community and health outcomes used to validate the vulnerability metrics across relevant studies. A narrative synthesis is more suitable for synthesizing more diverse evidence, allowing for concept mapping to advance theoretical development [20]. Based on its functions, we primarily aimed to synthesize components of community vulnerability and its indicators across relevant studies. The components of community vulnerability would be mapped based on the framework of the coupled human-environment system for vulnerability analysis. Additionally, we intended to discuss the identified vulnerability components and how they interact and work to shape community vulnerability to a pandemic. As research on community vulnerability in the pandemic context grows, methodological problems also raise concerns, ranging from index construction to validation (similar to issues seen in the context of heat stress) [21]. This can hinder the advancement of relevant research and policymaking using vulnerability indices. Therefore, another objective of this systematic review was to identify any methodological shortfalls in the construction and validation of community vulnerability indices relevant to a pandemic.

## METHODS

This study was pre-registered with Prospective Register of Systematic Reviews (Reference No.: CRD42021266186) and prepared following the Preferred Reporting Items for Systematic Review and Meta-Analysis (PRISM) [20].

### Search strategy and selection criteria

We targeted studies focusing on community vulnerability in the context of a pandemic or emerging infectious diseases (EIDs) caused by novel respiratory viruses and qualifying as pandemic. Literature was retrieved from five databases (PubMed, Embase, Web of Science, PsycINFO, and Scopus) to encompass multiple disciplines. The literature search was performed in December 2021 to capture all articles published up to November 30, 2021, using a combination of search terms related to community vulnerability and emerging infectious diseases (EID) that are caused by novel respiratory viruses. We limited the review to EIDs caused by novel respiratory viruses because these diseases share similar (if not common) transmission modes, control measures, and clinical consequences, thus sharing similar indicators of vulnerability and operations of the systems to shape community vulnerability. Due to the high heterogeneity of terms relating to community vulnerability, we performed a backward/forward literature search, title, abstract, and full-text screening to cover a list of search terms related to community vulnerability as exhaustively as possible. A full list of search terms was shown in Table S1 in the **Online Supplementary Document**.

**Figure 1:**
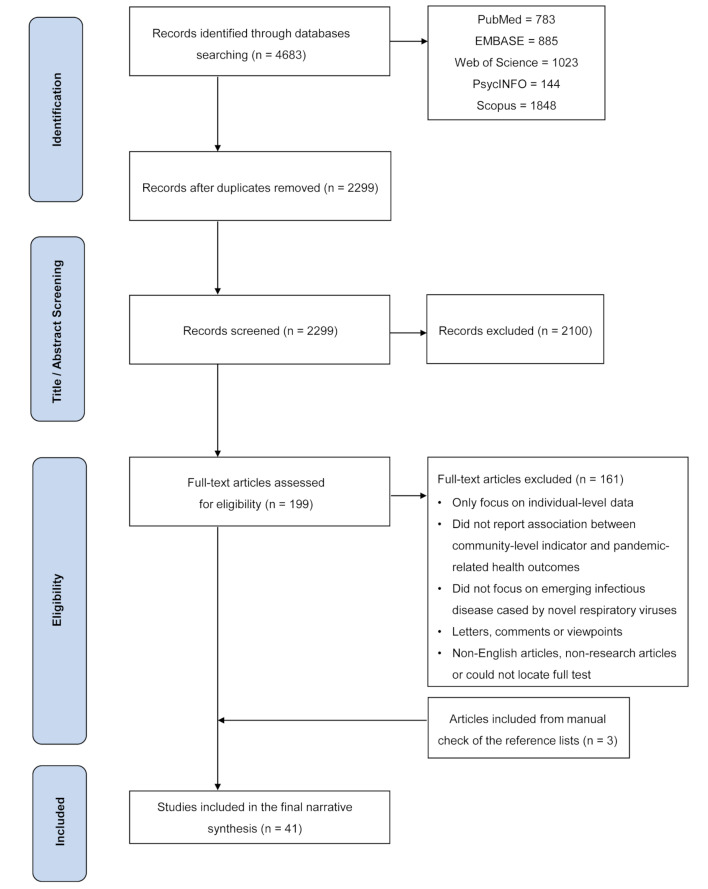
A flowchart of article identification, screening, inclusion, and exclusion.

After deduplication, two researchers independently screened the titles and abstracts of retrieved articles based on the inclusion and exclusion criteria (Table S1 in the **Online Supplementary Document**). We included empirical studies that focused on constructing a composite vulnerability index or used multiple indicators to evaluate and map community vulnerability on a geographic unit basis. To be included, studies also had to report the associations between the vulnerability levels and at least one morbidity outcome (disease incidence rates, case counts), mortality outcome (mortality rates, death counts and case-fatality ratio) of a defined period, or changes of these health outcomes by a specific time unit. Then, two researchers independently screened the full texts of potentially eligible articles. Finally, the reference lists of all eligible articles were manually screened to retrieve additional articles that were not captured in electronic screening. The process flowchart for article identification, screening, inclusion, and exclusion is shown in **Figure 1**.

### Data synthesis

We used common narrative synthesis tools for data synthesis, including tabulation, thematic analysis, vote counting, and concepting mapping [20]. Two researchers independently extract information from the eligible articles using a standard table, including the following: authors, year of study, study site, geographic unit of the defined community and number of the geographic units, components and/or indictors of community vulnerability, methods used to construct vulnerability indices, health outcomes used to validate the vulnerability indices/indicators, and the associations of health-related outcomes with the vulnerability indices and/or indicators, as well as the statistical approaches used to reach the conclusions. A third researcher cross-checked all the extracted data by reviewing the full texts of all eligible articles. We conducted a thematic analysis to code the extracted indicators and components of community vulnerability identified from the included studies. Two researchers conducted the coding independently, and a third researcher joined to co-determine the final list of community vulnerability components (or themes) and their indicators. We used vote counting as described by Popay et al. [20] to count the frequency of each community vulnerability indicator used among the included studies and its reported associations with the morbidity or mortality outcomes across the studies. Vote counting provided a preliminary analysis of the associations of community vulnerability and their indicators with the morbidity or mortality outcomes, which served as a basis for exploring the differences within and between studies [20]. Finally, we used the framework of the coupled human-environment systems for vulnerability analysis [18] to map the inter-relationships of the identified vulnerability components, which in turn represented a pathway to advance the development of the vulnerability framework in the pandemic context.

### Quality assessment

To assess the methodological quality of each included study, we used a checklist adapted from a previous systematic review evaluating the methodological approaches of the construction and validation of community vulnerability indices related to heat [21]. Details of the quality assessment for each article based on the checklist are available in Table S2 in the **Online Supplementary Document**.

## RESULTS

### Characteristics of the included studies

A total of 41 studies were included in the analysis (**Table 1**). All the included studies focused on community vulnerability to COVID-19. Of the 41 studies, 27 were conducted in the United States, eight in Brazil, one each in Australia, China, England, India and Nigeria, respectively, with the remaining one focusing on a global region covering 156 countries. The reported geographic units of analysis ranged from neighbourhood (eg, census tract, zip code, community area, middle layer super output areas, or local government area), to county (or municipality), state, and country level.

**Table 1:** Characteristics of articles included for this narrative synthesis

Authors & year	Study site	Geographic unit (number of units)	Components and indicators of community vulnerability index	Methods to construct vulnerability index	Health outcomes used to validate the vulnerability levels	Statistical approach to validate the vulnerability levels
Al Rifai et al., 2021 [22]	USA	State	CDC’s SVI. Socioeconomic status: poverty, unemployment, income, education; household composition and disability: elderly, children, disability, single parents; Minority status and language: minority, language proficiency; Housing and transportation: multiunit buildings, mobile homes, household crowding, households without a car, group quarters.	Percentile rank and equal weight assigned for indicators and domains.	Cumulative COVID-19 incidence rate and mortality rate as of August 19, 2021.	Pearson correlation and linear regression.
Amram et al., 2020 [23]	Washington State, USA	Zip code (n = 112)	Race/ethnicity, chronic conditions, vulnerable occupations, poverty, population density.	The standardized coefficients of the indicators from the multilevel modelling were used to create the composite index.	Cumulative COVID-19 incidence rate as of June 10, 2020.	Multilevel modelling to accommodate the cluster effect of county.
Arsalan et al., 2020 [15]	Global	Country (n = 156)	InfoRM index: natural hazards, human hazards, social development and deprivation, inequality, aid dependency, vulnerable groups, institutional capacity, communication infrastructure, physical infrastructure, health care systems; Infectious disease vulnerability index: demographic, health care, public health, disease dynamics, political domestic, political international, economic; Elderly ratio; Policy stringency index: an index generated by combining 13 COVID-19 control policies.	Composite scores of relevant domains or indicator scores (no clear information about how to generate the composite indices).	Cumulative COVID-19 incidence rate and mortality rate by six intervals between January 22, 2020, and May 11, 2020.	Multiple regression with multicollinearity pre-evaluated before running the regression.
Baggio et al., 2021 [24]	Alagoas, Brazil	Municipality (n = 102)	Brazil’s SVI. Urban infrastructure: household sanitation facilities, neighbourhood sanitation facilities, socially disadvantaged population; Human capital: infant mortality, children’s school attendance, underage mothers, single parents, illiteracy rate, education, socially disadvantage population (aged 15-24); Income and work: income, unemployment, activity rate. Other indicators or covariates: Gini index, public sector workers.	No information about how to generate the index.	Cumulative COVID-19 incidence rate and mortality rate; case-fatality ratio between March and August 2020.	Moran’s bivariate spatial correlation.
Biggs et al., 2021 [25]	Louisiana, USA	Census tract (n = 1105)	CDC’s SVI. Other indicators or covariates: population density.	Percentile rank and equal weight assigned for indicators and domains.	Cumulative COVID-19 incidence rate from March 9 to August 24, 2020.	Negative binomial regression.
Bilal et al., 2021 [26]	New York, Philadelphia and Chicago, USA	ZIP code (n = 58, 177, and 46 in Chicago, New York and Philadelphia, respectively)	CDC’s SVI. Other indicators or covariates: elderly rate.	Percentile rank and equal weight assigned for indicators and domains for generating SVI which was then standardized by city mean.	COVID-19 testing rate; Test positivity ratio; Cumulative COVID-19 incidence rate and mortality. rate from March to September 2020.	Spatial conditional autoregressive negative binomial model for each city.
Castro et al., 2021 [27]	Brazil	Municipality (n = 5565)	Brazil’s SVI. Additional indicators or covariates: illiteracy rate, Gini index, income, household crowding, household sanitation facilities, population density, primary health care capacity.	No information about how the composite index was generated.	Cumulative COVID-19 incidence rate and mortality rate between February 26 and July 31, 2020.	Geographic weighted regression analysis.
Credit K, 2020 [9]	Chicago & New York, USA	Zip code	Race/ethnicity, income, population density, physical activity facilities, health care facilities, food accessibility, elderly, health care manpower, household crowding, COVID-19 testing	No composite index. Indicator scores were used.	Cumulative COVID-19 incidence rate	Linear regression and exploratory spatial analysis.
Daras et al., 2021 [28]	England	Middle layer super output areas (n = 6789)	Hospital admissions due to chronic conditions, health care infrastructure, race/ethnicity, household crowding, poverty.	Standardized individual indicator scores.	Age-standardized mortality rate due to COVID-19 between 1 Mar-31 May 2020	Multivariable general estimating equation and Poisson regression.
Dasgupta et al., 2020 [29]	USA	County (n = 3142)	CDC’s SVI. Additional indicators or covariates: urbanicity/rurality	Percentile rank and equal weight assigned for indicators and domains.	Risk of becoming COVID-19 hotspot in June to July, 14 d after initial hotspot identification.	Bivariate log-binomial models. Analyses were stratified by urbanicity.
De Souza et al., 2020 [10]	Brazil	Municipality (n = 2496)	Brazil’s SVI.	No explanation for how SVI and their domain indies scores were constructed.	Cumulative COVID-19 incidence rate and mortality rate as of May 6, 2020.	Ordinary least square regression and spatial regression.
Frisina Doetter, et al.2021 [30]	USA	State (n = 50)	CCVI. Socioeconomic status: poverty, unemployment, income, education, (health) insurance coverage; Minority status and language: Race/ethnicity, Language proficiency; Household and transportation: household crowding, households without a car, children, single parents, household sanitation facilities, mobile homes, disability; Epidemiological factors: chronic conditions (5 indicators), elderly; Healthcare system factors: health system capacity, health system strength, health care accessibility, health system preparedness; High risk environments: vulnerable occupations; Population density; Policy stringency.	Percentile rank and equal weight assigned for indicators and domains.	Age-and ethnicity-standardized mortality rate due to COVID-19 as of December 2020.	Pearson correlations and multiple linear regression.
Gorris et al, 2021 [31]	New Mexico, USA	County (n = 33)	Population size, poverty, household crowding, race/ethnicity.	Generalized propensity score to calculate the time-varying vulnerability based on the indicators and the weekly number of cumulative COVID-19 case counts.	Cumulative COVID-19 death counts and the case-fatality ratio from April to August 2020.	Spearman correlation.
Huang et al., 2021 [32]	South Carolina, USA	County (n = 46)	SVI. Race/ethnicity, single parents, property, income, rent burden, age, household crowding, dependency, social security benefits, language proficiency, hospital per capita, nursing home residents, vulnerable occupation, unemployment, sex.	PCA to extract main components then equal weight assigned to each component to generate percentile rank for overall vulnerability.	Cumulative COVID-19 incidence rate and mortality rate of two stages: March to May 2020 and June to September, 2020.	Univariate correlation analyses and ordinary least square regression.
.Islam et al., 2021 [33]	USA	County (n = 3140)	CDC’s SVI.	Percentile rank and equal weight assigned for indicators and domains.	Cumulative COVID-19 incidence rates and mortality rates as of August 10, 2020.	Poisson Regression.
Islam et al. 2021 [34]	USA	County (n = 3091)	CDC’s SVI. Racial composition from SVI: race/ethnicity. Other indicators or covariates: elderly, COVID-19 testing, chronic conditions, air quality, air temperature, precipitation.	Percentile rank and equal weight assigned for indicators and domains.	Cumulative and weekly COVID-19 incidence rate and mortality rate from March 22, 2020, to March 6, 2021.	Negative-binomial mixed-effects model.
Jackson, et al. 2021 [35]	USA	County (n = 3140)	SVI: house value, income, rent burden, race/ethnicity, age, female-headed households, poverty, unemployment, education, households without a car, single parents, social security benefits, household crowding, renters, language proficiency, (health) insurance coverage, health care facilities, population density, vulnerable occupations, unemployment/employment (female), sex ratio. Other indicators or covariates: COVID-19 control policies, urbanicity/rurality	PCA to extract main components which were then summed to generate the overall vulnerability index.	Cumulative COVID-19 incidence rate and mortality rate from January 21, 2020, to January 30, 2021.	Spearman’s Correlation and spatial lag regression.
Karaye et al., 2020 [4]	USA	County (n = 2844)	CDC’s SVI. Other indicators or covariates: population size, population density, COVID-19 testing, climate conditions, PM_2.5_.	Percentile rank and equal weight assigned for indicators and domains.	Cumulative COVID-19 incidence rate between 21 Jan 2020 and 12 May 2020	Ordinary least squares regression and geographically weighted regression.
Karmakar et al., 2021 [36]	USA	County (All countries)	CDC’s SVI. Additional indicators or covariates: race/ethnicity, health insurance coverage, use of public transport, Gini index, food accessibility, health care infrastructure, life expectancy, obesity, population density, urbanicity/rurality, COVID-19 testing.	Percentile rank and equal weight assigned for indicators and domains.	Cumulative COVID-19 incidence rates and mortality rates from Mar 25 to Jul 29, 2020; and weekly change in incidence and mortality rate	Mixed-effects negative binomial regression (for incidence rate); zero-inflated negative binomial regression (for mortality rate).
Khazanchi et al., 2020 [5]	USA	County (n = 2754)	CDC’s SVI. Additional indicators or covariates: urbanicity/rurality, COVID-19 control policies.	Percentile rank and equal weight assigned for indicators and domains.	Cumulative COVID-19 incidence rate and mortality rate as of 19 Apr 2020	Population-weighted, quasi-Poisson regression.
Kiaghadi et al. 2020 [37]	Harris County, USA	Census tract (n = 786)	Household crowding, medical service utilizations, (health) insurance coverage, chronic conditions, health status, disability, age, exposure to pollutants, air quality or pollution, natural hazards, drinking, smoking, physical activity, obesity, sleep quality, education, poverty, living alone.	PCA to extracted five components which were then used to generate the overall vulnerability index and vulnerability rank using Rank-based exceedance method and Standard K-means cluster analysis, respectively.	Cumulative COVID-19 case counts as of August 16, 2020.	Spearman’s correlation.
Kim et al., 2020 [38]	Chicago, USA	Community Areas (n = 77)	Social vulnerability and health risk index: poverty, education, single parents, income, unemployment, race/ethnicity, chronic conditions, obesity, smoking.	PCA to extract two components – social vulnerability and health risk.	Cumulative COVID-19 mortality rate as of April 2020.	Bivariate correction and structural equation modelling.
Lawal et al, 2021 [39]	Nigeria	State (n = 20)	SVI: Elderly, mobility, urbanicity/rurality, GDP, health care facilities, population density, household sanitation facilities, poverty.	Each indicator score was standardized and summed to generate the composite vulnerability score.	Cumulative COVID-19 case counts and death counts by April 2020.	Bayesian correlation.
Lewis et al., 2020 [40]	Utah, USA	Utah small statistical areas (n = 99)	Health improvement index: income, income inequity, unemployment, poverty, single parents, illiteracy rate, education. Other indicators or covariates: race/ethnicity, food accessibility, (health) insurance coverage, household crowding, vulnerable occupations.	No clear information about how the composite score was aggregated but composite score was categorized into quintiles.	Age-weighted cumulative COVID-19 incidence rate, hospitalization rate, testing rate and test positivity rate during March 2 to July 9, 2020.	Binary logistic regression.
Liao et al. 2021 [41]	Hong Kong, China	District (n = 18)	CCVI. Socioeconomic status: poverty, unemployment, income, education; Household composition: elderly, children, single parent, elderly living alone; Housing condition: household crowding; Healthcare system factors: health care facilities and manpower; Epidemiologic factors: population density, obesity, hypertension, smoking, vulnerable occupations, mobility, entertainment venues, race/ethnicity.	Percentile rank and equal weight assigned for indicators and domains.	Cumulative COVID-19 case counts from January 23 to August 31, 2020.	Pearson correlation and Poisson regression.
Martins-Filho et al., 2020 [42]	Aracaju municipality, Brazil	Neighbourhood	Living condition index: illiteracy rate, income, poor housing conditions.	Indicator scores were summed to generate the composite score which was grouped into quartiles.	Cumulative COVID-19 incidence rate and case-fatality ratio as of July 14, 2020.	Cochran-Armitage test.
Martins-Filho, et al., 2021 [14]	Brazil	State (n = 27)	Brazil’s SVI. Additional indicators or covariates: Gini index.	No information about how the SVI index was generated.	Cumulative COVID-19 incidence rates and mortality rates in children aged 0-19 y and case-fatality ratio as of 3 Sep 2020	Spearman's rank correlation.
Moise, 2020 [43]	Miami-Dade County, USA	Census block group level (n = 1594)	Socioeconomic status and socioeconomic status opportunity index: poverty, income, language proficiency, private vehicle, mobile home, rent burden, disability, (health) insurance coverage. Social disadvantage index: language proficiency, single parents, children, elderly, education, (health) insurance coverage. Convergence of vulnerability index: poverty, disability, children, (health) insurance coverage, race/ethnicity, unemployment.	PCA to extract the three indices. No detailed explanations for how to generate the composite index scores.	Cumulative COVID-19 incidence rate as of July 21, 2020.	Ordinary least square regression.
Neelon et al., 2021 [7]	USA	County (n = 3142)	CDC’s SVI. Additional indicators or covariates: rurality, health status, sex, smoking, air quality, health care manpower, air temperature, precipitation, COVID-19 testing.	Percentile rank and equal weight assigned for indicators and domains.	Daily incidence and mortality rates of COVID-19 by stages from March 15 to December 31, 2020.	Bayesian hierarchical negative binomial regression.
Oates, et al., 2021 [44]	Alabama and Louisiana, USA	Census tract (n = 1160 in Alabama and n = 1105 in Louisiana)	CDC’s SVI. Additional indicators or covariates: rurality, population density.	Percentile rank and equal weight assigned for indicators and domains.	Test rate for COVID-19; cumulative COVID-19 incidence rate; test positive rate from February 27 to October 7, 2020.	Multivariable negative binomial regression.
Ossimetha et al., 2021 [45]	USA	County (n = 2664)	Social deprivation index: education, single parents, household crowding, mobile home, private vehicle, unemployment, poverty. Additional indicators or covariates: population size, population density, urbanicity/rurality.	A composite score was generated but no information about how to generate the score.	Growth in COVID-19 incidence rate and mortality rate in May 2020 relative to April 2020	Linear regression.
Raymundo, et al., 2021 [46]	Brazil	Municipality (n = 5570)	Population size, elderly, minority/ethnicity, health care capacity/resources, income, Gini index, unemployment, mortality, human development index, education, household sanitation facilities, poverty.	No information about how to generate composite indices.	Cumulative COVID-19 incidence rate from February 25 to September 26, 2020.	Spearman’s correlations; ordinary least square regression; geographically weighted regression
Rocha, et al., 2021 [47]	Brazil	State and municipality	Chronic conditions, obesity, smoking, household sanitation facilities, informal employment, income, education, health care infrastructure, health care manpower, social security/health benefits (eg, Bolsa Familia), physical distancing behaviours.	PCA to identify the most important components to indicate vulnerability.	Age-standardized monthly COVID-19 mortality rate from February 1 to October 31, 2020.	Pearson correlation; panel regression.
Saghapour et al, 2021 [48]	Victoria and New South Wales, Australia	Local government areas (n = 563)	CPVI. Socio-demographic: elderly, household crowding, socio-economic status; Medical conditions: deaths due to chronic diseases, flu/pneumonia-related deaths; Transportation: public transit use for work; transit stop density; Land use: population density; physical infrastructure (commercial areas, education centres, medical services).	PCA to extract and generate each domain score which was then summed to generate the overall CPVI with equal weight assigned to each domain.	Population-standardized COVID-19 case counts by May 3, 2020.	Correlation analysis.
Sarkar & Chouhan, 2020 [11]	India	County	Social ecological vulnerability index. Environment condition: PM_10_, PM_2.5_, NO_2_ and SO_2_; Econo-demographic: household crowding, population density, poverty, homeless; Social demographic: unemployment, race/ethnicity, socially disadvantage population; Geriatric health index: elderly, illiteracy rate, health care infrastructure, sex.	PCA to extract four components which were used to aggregate the overall vulnerability index; weight assigned based on the explained variance of each component.	Cumulative COVID-19 case counts and death counts as of June 25, 2020.	Person's correlation.
Siqueira et al., 2021 [49]	Brazil	Municipality (n = 5570)	Brazil’s SVI. Additional socioeconomic indicators: Gini index, unemployment, illiteracy rate, household sanitation facilities, poverty. Additional hospital services indicators: health care infrastructure and manpower.	Factor analysis to extract five factors based on the indicators.	Incidence rate and maternal mortality rate as well as case-fatality ratio due to COVID-19 from March 2020 to June 2021.	Spearman correlation; multivariate regression.
Snyder & Parks, 2020 [12]	USA	County (all counties in the USA)	Socio-ecological vulnerability index. Ecological factors: age, sex, population density, air quality, air temperature, humidity, connectivity (or human mobility); Health factors: smoking, diabetes, hypertension, obesity; Social factors: (health) insurance coverage, education, race, health care infrastructure; Economic factors: poverty, Gini index, vulnerable occupations (economic impact), vulnerable population (infection).	Percentile rank and equal weight assigned for indicators and domains.	Cumulative COVID-19 incidence rate as of September 11, 2020.	Ordinary least square regression and geographically weighted regression.
Tiwari et al., 2021 [50]	USA	County (n = 3142)	CCVI, CDC’s SVI. Epidemiological factors: chronic health conditions, obesity, population density, flu and pneumonia-related deaths. Healthcare system factors: health system capacity, health system strength, health system preparedness.	A machine learning method to generate a C19VI which was compared against the CCVI generated based on the CDC’s methods.	Daily change in case-fatality rate due to COVID-19, from January 22 to July 31, 2020; Secondary outcome: daily change in case counts and death counts.	The Receiver Operating Characteristic-Area Under the Curve to validate the prediction accuracy of C19VI. Friedman test, a two-tailed Wilcoxon signed rank test, and Boruta parameter importance assessment technique to compare C19VI and CCVI.
Yellow Horse, et al., 2020 [51]	New Mexico, USA	Zip code (n = 366)	CDC’s SVI. Historically embedded vulnerability: household communication facilities, household sanitation facilities, living in poor conditions, neighbourhood land pollution. Other indicators or covariates: population density, (health) insurance coverage.	Percentile rank and equal weight assigned for indicators and domains.	Cumulative COVID-19 incidence rate as of as of August 9, 2020.	Ordinary least square regression.
Yellow Horse et al, 2021 [52]	Arizona, USA	Zip code (n = 385)	Concentrated disadvantage index: poverty, female-headed households, socially disadvantage population, unemployment, education, income. Additional indicators or covariates: race/ethnicity, population density, elderly, (health) insurance coverage, household crowding, households without kitchen, households without plumbing, mobility, use of public transport, language proficiency, Gini index.	PCA to extract the factor of concentrated disadvantage and regression to generate the composite index score.	Cumulative COVID-19 case counts.	Poisson regression and geographically weighted Poisson regression.
Wang et al., 2020 [6]	USA	County	CDC’s SVI.	Percentile rank and equal weight assigned for indicators and domains.	Cumulative COVID-19 incidence rate and mortality rate as of July 21, 2020.	Spearman’s rank correlation.

CDC – United States Centers for Disease Control and Prevention, SVI – social vulnerability index, PCI – principal component analysis, InfoRM – index for risk management, C19VI – COVID-19 vulnerability index, CPVI – COVID-19 pandemic vulnerability index, CCVI – COVID-19 vulnerability index

### Methodological quality evaluation

Sixteen studies were rated to have fair quality (Table S2 in the **Online Supplementary Document**) [5,9-11,14,23,24,27,28,32,35,40,42,45,46,52], none of which provided information about the contribution of the chosen indicators to the overall vulnerability. Nine of these studies did not explain how the composite vulnerability indices were generated [9,10,14,23,24,27,40,45,46] and two [45,52] did not justify why the vulnerability indicators were chosen. Three studies [9,23,28] used individual vulnerability indicator scores directly rather than constructing a composite vulnerability index. Of the 38 studies that constructed a composite vulnerability index, 16 used percentile-rank methods (eg, the CDC’s SVI) [4-7,12,22,25,26,29,30,33,34,36,41,44,51] which assumed equal contribution of the chosen indicators and components to the overall vulnerability, 11 used principal component analysis (PCA) or factor analysis [11,32,35,37,38,43,47-49,52] to explore main components of vulnerability from the chosen indicators and assign weights to each component regarding their contribution to the overall vulnerability, two [39,42] directly summed the indicator scores, and two [31,50] used the more sophisticated methods (ie, machine learning, generalized propensity modelling) to generate an overall vulnerability index. Thirteen studies provided insufficient justifications about the chosen statistical methods used to examine the associations between the vulnerability levels and health-related outcomes, and nine [6,11,14,24,31,37,39,42,48] only examined their univariate correlations.

### Vulnerability indicators and components

A total of 48 vulnerability indicators of community vulnerability were extracted and categorized into 11 vulnerability components: socioeconomics and demographics, health condition and lifestyle behaviours, household composition, housing condition, health care resources, social and human development, urbanization and urban-built environments, race/ethnicity and language, policy and public response, viral exposure facilitators, and natural environments (**Table 2**). Of the 38 studies that used a composite vulnerability index, 13 used the CDC’s SVI [4-7,22,25,26,29,33,34,36,44,51], five used Brazil’s SVI [10,14,24,27,49], three used the Surgo Foundation’s CCVI [30,41,50], and the remaining used indices constructed using a heterogenous array of indicators.

**Table 2:** Indicators and components of community vulnerability

Vulnerability components	Vulnerability indicators	Frequency of the indicators included in the studies
Socioeconomics & demographics	Low income	31
	Unemployment	31
	Poverty	30
	Low education	29
	Age	28
	Female	5
Health condition & lifestyle behaviours	Chronic diseases	12
	Obesity	7
	Poor lifestyle behaviours	6
	Poor health status	3
Household composition	Single parent	28
	Disability	17
	Underage mothers	5
	Female-headed households	3
	Elderly living alone	2
Housing condition	Household crowding	28
	Mobile home, renters or homeless	19
	Households without a car	17
	Household sanitary facilities	11
	Other household facilities	3
	Instituting housing	1
Healthcare resources	Healthcare facilities (eg, hospitals, hospital beds, ICU beds)	9
	Health insurance coverage	9
	Healthcare manpower (eg, physicians, nurses)	6
	Healthcare system (preparedness, strength, accessibility)	6
	Primary health care and public health resources	5
Social and human development	Economic inequity	10
	Literacy	8
	Life expectancy/infant mortality	6
	Social security benefit	3
	GDP	1
Urbanization and urban built environments	Urban infrastructure (public spaces, sanitation, communication)	8
	Urbanicity	8
	Residential segmentation by race/social status	6
	Food accessibility	3
Race/Ethnicity & language	Ethnical minority	29
	Language unproficiency	19
Policy and public response	COVID-19 testing	5
	COVID-19 control policy stringency	4
	New ICU beds	2
	Public physical distancing compliance	1
Viral exposure facilitators	Population density	20
	Population mobility and connectivity	11
	High-risk occupations	8
	Risk venues (eg, catering, entertainment)	1
Natural environments	Climate conditions	6
	Air pollution	6
	Land pollution	2

### Associations of community vulnerability levels with COVID-19 morbidity and mortality

Twenty-six of the 29 studies reporting the associations of a composite vulnerability index with measures of cumulative COVID-19 morbidity reported a significant association between the two [4-6,10,11,15,22-27,32-37,39,40,42-44,48,49,52]. Three studies reported that COVID-19 cases or incidence rates initially rapidly increased in less vulnerable communities, but eventually became more widespread in more vulnerable communities [7,32,41]. Nineteen of the 21 studies measuring cumulative mortality outcomes reported significant associations between a higher composite vulnerability score and higher COVID-19 mortality [5,6,11,14,15,22,26-28,31-36,38,39,43,47,49]. One study indicated that community vulnerability levels were initially negatively associated with COVID-19 mortality, with the association becoming positive when the pandemic remitted, and then reverting to negative once more during a subsequent new growth phase [7]. Another study conducted in the United States, however, indicated that the vulnerability level was positively associated with COVID-19 mortality in Black and Hispanic people, but not in White people [30]. Six studies assessed the associations of community vulnerability levels with changes in COVID-19 morbidity and/or mortality outcomes [29,34,36,45,47,50]. All studies concluded that initial clusters of cases or deaths tended to be reported in less vulnerable communities, but more vulnerable communities had greater risk of becoming a subsequent COVID-19 “hotspot” [29] or of having greater increases in incidence rates and/or mortality rates [34,36,45,47,50].

### Associations of vulnerability indicators with COVID-19 morbidity and mortality

Socioeconomic and demographic indicators (including poverty, low education, unemployment, and female gender) were consistently associated with higher COVID-19 morbidity or mortality. The associations of income and age with COVID-19 morbidity and mortality were more heterogeneous across studies. Poorer health conditions, including greater prevalence of chronic diseases [23,28,37,38], obesity [36,38,41], poor health status [7], and poor lifestyle behaviours [37,38], were associated with higher COVID-19 morbidity and mortality.

For household composition, only single-parent household was positively associated with both COVID-19 morbidity and mortality [36]. Regarding housing condition, household crowding [9,27,28,36,41,51,52], and mobile home and instituting housing [36] were associated with higher COVID-19 morbidity and mortality. Households without a car [36,51] and with insufficient sanitary facilities [27,46,49,51,52] (for example, without running water) were associated with greater COVID-19 morbidity, but not mortality.

Healthcare resources, including primary health care and public health resources [7,36,46,47,49] and health insurance coverage [12,33,36,51,52], were consistently associated with lower COVID-19 morbidity and mortality, while hospital resources (eg, hospital and intensive care unit (IC) beds and physicians) had inverse associations with COVID-19 morbidity and/or mortality [9,28,36,41,46,47,49]. Two studies [27,37], however, reported that communities with greater primary health care coverage had more COVID-19 cases, possibly due to greater accessibility to viral testing and capacity to diagnose cases.

More advanced social and human development, indicated by lower economic inequity, greater life expectancy, higher gross domestic product, and higher literacy rates were associated with higher COVID-19 morbidity [10,12,27,36,46,49], but lower mortality [14,27,36,49]. The urban-built environments with better urban infrastructure (such as spaces for physical activities and sanitation infrastructure [9,49,51] and food accessibility [36]) were associated with lower COVID-19 morbidity and mortality, while living in residential segmentations [9,51] was associated with higher COVID-19 morbidity. Urbanicity had heterogeneous associations with both COVID-19 morbidity [5,7,32,35,36] and mortality [7,32,35] across studies. Ethnic minority membership and limited proficiency in native/official languages were consistently associated with higher COVID-19 morbidity [4-6,9,12,23,25,26,34,36,44,46,51,52] and mortality [5,6,26,28,31,33,34,36,38,50,52]. However, the prevalence of non-Chinese people who tended to be better educated, was associated with lower COVID-19 morbidity in Hong Kong [41].

Viral exposure facilitators (including greater population density [12,23,25,27,36,41,46,52], population mobility and connectivity [10,36,41,52], and prevalence of people working in high-risk occupations [23] and risk venues[41]) were associated with higher COVID-19 morbidity. Greater population density was also associated with higher COVID-19 mortality [27,36]. For natural environments, a specific range of humidity [12] and low temperature [7], poorer air quality [7,12,37], and land pollution [37] were associated with higher COVID-19 morbidity, while low temperature and air pollution were also associated with greater mortality [7].

Policy responses, including more COVID-19 testing [7,9] and adding new ICU beds [49], were correlated with increases in COVID-19 incidence [7,9,49] and mortality [7,49], indicating greater policy response to the increased stress caused by the pandemic. More stringent policies to limit population mobility and contacts were associated with both lower COVID-19 morbidity and mortality [35]. However, another study found that more stringent policies were associated lower COVID-19 incidence rate in the early pandemic stage, but not in the later stage [15], possibly because social distancing behaviours had been widespread in the later pandemic stage. One study conducted in the US found that more stringent policies were associated with lower COVID-19 mortality in While people but greater mortality in Black people [30], indicating that Black people who tended to take on the low-wage frontline essential jobs did not benefit from stringent policies for controlling COVID-19.

## DISCUSSION

**Figure 2:**
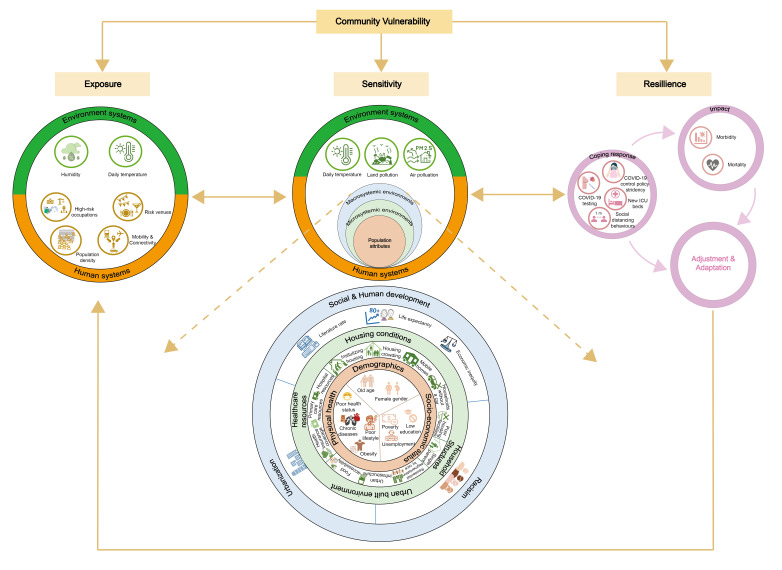
Community vulnerability to a pandemic based on the coupled human-environment system.

The framework for vulnerability analysis based on the coupled human-environment systems [18] was used to facilitate our integration of the vulnerability components identified and our discussion on their interactions and interdependence through which they shape community vulnerability (**Figure 2**). Based on the framework, vulnerability is manifested in three components: exposure, sensitivity, and resilience. Exposure is characterized by frequency, magnitude, and duration of hazard exposure. Sensitivity is the degree to which the systems can be modified by disturbances and stresses, being characterized by available biophysical and social resources, while resilience refers to adaptive capacity, the ability of the systems to bounce back or evolve into new equilibriums. These three components are interdependent, with feedbacks from one component working back upon the other two.

For exposure, environmental factors of a specific range of low temperature and humidity can prolong and intensify exposure [7,12] because they are associated with increased virus stability and survival time [53]. However, meteorological factors do not act alone, but rather interact with the human systems. Important factors of the human systems that sustained viral transmission and duration of exposure included population density [12,23,25,27,36,41,46,52], human mobility and connectivity [10,36,41,52], occupational exposure [23,41], and risk settings [41] that accommodate human demands for services (eg, catering), entertainment (eg, bars), and social interactions. These human factors are inextricably intertwined with the intensification of globalization, urbanization, economic connectivity and activity, and with the growth in consumerism [54,55]. The viral exposure facilitators explained why COVID-19 was usually first introduced and widely seeded in more urbanized areas by the more socially privileged groups [29,34,36,45,47,50] through their social, economic, and entertainment activities.

Once viruses enter a community, a pandemic’s impact is determined by system sensitivity. Air pollution [7,13,46], land pollution [46], and low temperature [13] were the identified environmental factors that can predispose the population to greater vulnerability to COVID-19 mortality. Air pollution and cold temperature can compromise humans’ innate immune defence system, induce additive or synergistic inflammatory response to the infection, and aggravate pre-existing chronic diseases [56]. Within the human systems, sensitivity is determined by the population’s biophysical capacity and social resources shaped by interdependent and hierarchical vulnerability components: the inner circle represents the population attributes encompassing demographics, socioeconomics, and health condition; the middle circle represents the microsystemic environments, including housing conditions, household composition, access to health care resources, and the neighbourhood environments, while the outermost layer represents the more macrosystemic environments, including social and human development, urbanization, and (mostly structural) racism (**Figure 2**). The microsystemic environments are influenced by and influence the population attributes. Socially disadvantaged populations tend to live in overcrowding households with insufficient sanitary facilities and reside in neighbourhoods with limited access to health care resources, food, sanitation, green spaces, and other community resources [57], with the expansion of megacities [58]. Conversely, poor living conditions and neighbourhood environments can compromise defensive immune response and accelerate viral transmission within households and neighbourhoods [55], and thereby erode health conditions and exacerbate poverty. Improved primary health care and health insurance can enhance population health conditions and are thereby important for reducing COVID-19 morbidity and mortality [7,12,33,36,46,47,49,51,52] in countries lacking social medical systems. The macrosystemic environments further exert influences on the sensitivity of the macrosystemic environments and population attributes. Uneven socioeconomic development and rapid urban expansion is compound with inappropriate or inadequate urban infrastructure [55,59]. Structural racism perpetuate inequalities of wealth, social protection, and access to food, health care and education [60,61], and deprive dense urban population particularly racially and ethnically marginalized groups of green and blue spaces [61]. Such systems compress urban growth, forcing it outwards in ways that eventually disturb the ecological environments and exacerbate pollution and climate change [62]. Structural inequalities fuel pandemic crisis which in turn, exacerbates inequalities. This encourages rethinking about pathways to goals of sustainable social and human development [63].

System resilience is rooted in the human systems that shape human biophysical capacity and access to social resources. Institutional response including increasing diagnostic testing and adding new ICU beds were the main response to stress placed onto the systems by the pandemic [7,9,49]. However, power asymmetries privileging wealthy groups in access to these health care resources can widen health inequalities [64], while restructuring policies to empower the more vulnerable communities in access to COVID-19 diagnostic testing can reduce them [44]. Stringent policies to limit population mobility were suggested for reducing COVID-19 morbidity and mortality [13], but the effect of these was more prominent in the early pandemic stages [65]. Prolonged social distancing measures disproportionately burdened the mental well-being and access to life necessities in more socially disadvantage populations [66], and hence increased rather than reduced COVID-19 mortality in these populations [40]. The coping response and pandemic impacts eventually resulted into system adjustment and adaptation, which act back upon to increase/reduce the systems’ exposure and sensitivity. However, a primary focus on returning to normal business, driven by economic forces rather than building better systems, increases the probability of repeated pandemic resurgence and represents a prolonged problem for the systems [67]. Technological and digital transformation and high vaccination uptake are important to facilitate system adaption and evolution into a new balance [68].

We identified major shortfalls of the included studies on community vulnerability to a pandemic. First, these studies primarily viewed community vulnerability as the absence of entitlements among which the interrelationships and interdependence with the environments were largely dismissed. Second, justifications for the chosen community vulnerability indicators were generally insufficient across the included studies. A few studies chose indicators based on data availability and used PCA to screen important indicators and classify them into components (factors) [11,32,35,37,38,43,47-49,52]. Such data-driven methods can help address multicollinearity problems between indicators, but can also generate components (or factors) that may be difficult to interpret. Furthermore, most of the identified studies focused on community vulnerability in the early stage of the pandemic. The dynamic nature of community vulnerability as the pandemic unfolded was largely overlooked.

Our study has some limitations. First, as a narrative synthesis, our study may be criticized for its insufficient rigorousness and transparency for quantitative evidence synthesis when compared to a meta-synthesis. However, a narrative synthesis is more suitable for answering broader research questions, synthesizing mixed (qualitative and quantitative) evidence, and enabling theoretical development. Second, this review excluded articles that used one single indicator to evaluate vulnerability to a pandemic and articles that did not measure any disease-related morbidity or mortality outcomes, and thus cannot exhaustively identify all potential vulnerability indicators. Third, our article search was restricted to those studies containing key terms loosely relating to “community vulnerability” and may be insufficient to capture a high heterogeneity of environmental contexts that shape community vulnerability to a pandemic. Furthermore, most of the included studies were conducted in the USA or Brazil, which limited the generalizability of the research findings. Increasing the availability and accessibility of relevant data from global to national levels and to smaller geographic unit (such as neighbourhoods) can help stakeholders of different levels to evaluate and manage community vulnerability to future pandemics. Our study may also be subject to publication bias due to restricting to only English published articles.

## CONCLUSIONS

Community vulnerability to a pandemic is the vulnerability of the ecological systems shaped by the complex interactions between the human and environment systems. Environmental factors of suitable temperature and humidity and human factors indicative of more advanced social and human development co-determine community vulnerability to viral exposure. However, the impact of the pandemic was determined by the systems’ biophysical and social resources. Pandemic recovery should focus on building more sustainable and resilient systems rather than simply resuming prior economic activities.

## Additional material:


Online Supplementary Document
