# Supplementary material for: Community vulnerability to the COVID-19 pandemic: A narrative synthesis from an ecological perspective
Source: J Glob Health. 2022 Dec 3;12:05054. doi: 10.7189/jogh.12.05054 (PMC9719409; doi:10.7189/jogh.12.05054)
Supplement: Online Supplementary Document [file jogh-12-05054-s001.pdf]

ONLINE SUPPLEMENTARY DOCUMENT

Title: Community vulnerability to the COVID-19 pandemic: a narrative synthesis from an ecological perspective

Authors: Qiuyan Liao, Meihong Dong, Jiehu Yuan, Wendy Wing Tak Lam, Richard Fielding

Table S1 Key words for searching the literature, and inclusion and exclusion criteria

Search terms related to community vulnerability	"social vulnerability" OR "community vulnerability" OR "community susceptibility" OR "social susceptibility" OR "community resilience" OR "vulnerability index" OR "vulnerability indices" OR "vulnerability indicator*" OR "vulnerable metric*" OR "vulnerability level*" OR "vulnerability categor*" OR "overall vulnerability" OR "vulnerability map*" OR "susceptibility map*" OR "spatial disparities" OR "community disparities" OR "social disparities" OR "spatial inequity" OR "community inequity" OR "social inequity" OR "spatial inequality" OR "community inequality" OR "social inequality" OR "social deprivation*" OR "socio* vulnerability" OR "socio* susceptibility" OR "socio* disparities" OR "socio* inequity" OR "socio* inequality" OR "vulnerable communit*"
Search terms related to EIDs caused by novel respiratory viruses and qualify as pandemic	"pandemic*" OR "epidemic*" OR "infectious disease*" OR "communicable disease*" OR "outbreak*" OR "COVID-19" OR "COVID 19" OR "SARS-CoV-2" OR "coronavirus" OR "2019 novel coronavirus disease" OR "2019 novel coronavirus infection" OR "2019-nCoV" OR "SARS" OR "severe acute respiratory syndrome" OR "influenza" OR "MERS" OR "Middle East Respiratory Syndrome"
Inclusion criteria	1. Use a composite vulnerability index or used multiple indicators to evaluate and map community vulnerability on a geographic unit basis in the context of a pandemic or EIDs caused by novel respiratory viruses and qualifying as pandemic: severe acute respiratory syndrome (SARS), Middle East respiratory syndrome (MERS), influenza pandemics, and the current COVID-19 pandemic.<br>2. Report the associations between the vulnerability levels (composite indices and/or indicator scores) and at least one of the following health outcomes: disease incidence rates, case counts, mortality rates, death

	counts and case-fatality ratio of a defined period, or changes of these health outcomes by a specific time unit.
Exclusion criteria	1. Studies that focused on risk factors of infections or deaths due to the diseases at individual levels.<br>2. Studies that focused on the associations of one single indicator or one specific category of indicators (e.g., demographics or climate conditions) with risk of infections or deaths due to the diseases, 3. 3. Studies that focused on other hazards rather than EIDs caused by novel respiratory viruses.<br>4. Letters, comments or viewpoints that reported no empirical data, non-research articles, non-English articles, or articles without accessible full texts.

Table S2 Assessment of methodological quality of the included studies

Study (Authors, year)	Risk-of-bias by Questions *							Total	Quality
	Q1	Q2	Q3	Q4	Q5	Q6	Q7		
Al Rifai, et al., 2021	2	2	2	2	1	0	1	10	Good
Amram, et al., 2020	2	2	0	2	1	0	1	8	Fair
Arsalan, et al., 2020	2	2	2	2	1	0	1	10	Good
Baggio, et al., 2021	1	1	0	2	1	0	1	6	Fair
Biggs, et al., 2021	2	2	2	2	1	0	1	10	Good
Bilal, et al., 2021	2	2	2	2	1	0	2	11	Good
Castro, et al., 2021	1	1	0	2	1	0	2	7	Fair
Credit, 2020	2	1	0	2	1	0	2	8	Fair
Daras, et al., 2021	2	1	2	2	1	0	1	9	Fair
Dasgupta, et al., 2020	2	1	2	2	2	0	1	10	Good
De Souza, et al., 2020	2	1	0	2	2	0	2	9	Fair
Frisina Doetter, et al.2021	2	2	2	2	1	0	1	10	Good
Gorris, et al, 2021	2	2	2	2	1	0	2	11	Good
Huang, et al., 2021	2	1	2	2	1	0	1	9	Fair
Islam, et al., 2021a	2	1	2	2	1	2	1	11	Good
Islam, et al. 2021b	2	1	2	2	1	1	1	10	Good
Jackson, et al. 2021	2	1	2	2	1	0	1	9	Fair
Karaye, et al., 2020	2	2	2	2	2	0	2	12	Good
Karmakar, et al., 2021	2	2	2	2	2	0	1	11	Good
Kiaghadi, et al. 2020	2	2	2	2	2	0	2	12	Good
Khazanchi, et al., 2020	2	1	2	1	1	0	1	8	Fair
Kim, et al., 2020	2	2	2	2	1	0	1	10	Good
Lawal, et al, 2021	2	1	2	2	2	0	1	10	Good
Lewis, et al., 2020	2	1	0	2	1	0	1	7	Fair
Liao, et al. 2021	2	2	2	2	2	0	1	11	Good
Martins-Filho, et al., 2020a	1	1	0	2	1	0	1	6	Fair
Martins-Filho, et al., 2020b	1	1	2	2	1	0	1	8	Fair
Moise, 2020	2	2	2	2	1	0	1	10	Good
Neelon, et al., 2021	2	2	2	2	2	0	2	12	Good
Oates, et al., 2021	2	1	2	2	2	0	1	10	Good
Ossimetha, et al., 2021	1	0	0	2	1	0	1	5	Fair
Raymundo, et al., 2021	1	1	0	2	1	0	1	6	Fair

Rocha, et al., 2021	2	2	2	2	2	0	2	12	Good
Saghapour, et al, 2021	2	2	2	2	1	1	1	11	Good
Sarkar & Chouhan, 2020	1	2	2	2	1	0	1	9	Fair
Siqueira, et al., 2021	2	2	2	2	2	0	2	12	Good
Snyder & Parks, 2020	2	2	2	2	1	0	1	10	Good
Tiwari, et al., 2021	2	2	2	2	1	0	2	11	Good
Wang, et al., 2020	2	2	2	2	1	0	2	11	Good
Yellow Horse, et al., 2020	2	2	2	2	2	0	1	11	Good
Yellow Horse, et al., 2021	2	0	2	2	1	0	2	9	Fair

* Severn questions were constructed to evaluate the methodological quality of the included studies based on the study conducted by *Cheng et al. (2021). These seven questions were: Q1.* Was the construction of the vulnerability index referring to any related theory? Q2. Are all the indicators have justification from previous studies? Q3. Was there sufficient information about how the composite index was generated? Q4. Were the data sources clearly described? Q5. Were correlations between indicators to vulnerability and health outcomes reported? Q6. Were correlations between indicators and the composite vulnerability index reported? Q7. Was there sufficient justification about the chosen statistical analyses? Each included study was assigned a score of “2”, “1” or “0” when its risk was evaluated to be “definitely low”, “probably low” and “definitely high”, respectively, for each question. A summed score was finally generated based on evaluation of the studies for all the seven questions, which was then used to classify the study into poor (0-4), fair (5-9), good (10-14) quality.
